# The MKKK62-MKK3-MAPK7/14 module negatively regulates seed dormancy in rice

**DOI:** 10.1186/s12284-018-0260-z

**Published:** 2019-01-22

**Authors:** Xingxue Mao, Jianjun Zhang, Wuge Liu, Shijuan Yan, Qing Liu, Hua Fu, Junliang Zhao, Wenjie Huang, Jingfang Dong, Shaohong Zhang, Tifeng Yang, Wu Yang, Bin Liu, Feng Wang

**Affiliations:** 1 Guangdong Academy of Agricultural Sciences, Rice Research Institute, Guangzhou, 510640 China; 2 Guangdong Key Laboratory of New Technology in Rice Breeding, Guangzhou, 510640 China; 3 Guangdong Key Laboratory for Innovative Development and Utilization of Forest Plant Germplasm, College of Forestry and Landscape Architecture, SCAU, Guangzhou, 510642 China; 4 Agro-biological Gene Research Center, Guangdong Academy of Agricultural Sciences, Guangzhou, 510640 China

**Keywords:** Rice (*Oryza sativa* L.), *Pre-harvest* sprouting, Dormancy, MAPK cascade

## Abstract

**Background:**

Seed dormancy directly affects the phenotype of pre-harvest sprouting, and ultimately affects the quality and yield of rice seeds. Although many genes controlling seed dormancy have been cloned from cereals, the regulatory mechanisms controlling this process are complex, and much remains unknown. The MAPK cascade is involved in many signal transduction pathways. Recently, *MKK3* has been reported to be involved in the regulation of seed dormancy, but its mechanism of action is unclear.

**Results:**

We found that *MKKK62*-overexpressing rice lines (OE) lost seed dormancy. Further analyses showed that the abscisic acid (ABA) sensitivity of OE lines was decreased. In yeast two-hybrid experiments, MKKK62 interacted with MKK3, and MKK3 interacted with MAPK7 and MAPK14. Knock-out experiments confirmed that *MKK3*, *MAPK7,* and *MAPK14* were involved in the regulation of seed dormancy. The OE lines showed decreased transcript levels of *OsMFT,* a homolog of a gene that controls seed dormancy in wheat. The up-regulation of *OsMFT* in MKK3-knockout lines (OE/*mkk3*) and MAPK7/14-knockout lines (OE/*mapk7*/*mapk14*) indicated that the MKKK62-MKK3-MAPK7/MAPK14 system controlled seed dormancy by regulating the transcription of *OsMFT*.

**Conclusion:**

Our results showed that *MKKK62* negatively controls seed dormancy in rice, and that during the germination stage and the late stage of seed maturation, ABA sensitivity and *OsMFT* transcription are negatively controlled by *MKKK62*. Our results have clarified the entire MAPK cascade controlling seed dormancy in rice. Together, these results indicate that protein modification by phosphorylation plays a key role in controlling seed dormancy.

**Electronic supplementary material:**

The online version of this article (10.1186/s12284-018-0260-z) contains supplementary material, which is available to authorized users.

## Background

Pre-harvest sprouting (PHS) reduces the production levels of rice (*Oryza sativa* L.), wheat, maize, barley, sorghum, and soybean crops worldwide, leading to substantial agricultural losses. Therefore, it has attracted the interest of researchers in diverse fields (Fang and Chu 2008; Li et al. 2004; Simsek et al. 2014). To date, however, PHS remains a serious problem in agricultural production.

Seed dormancy is determined by genetic and environmental factors (Finkelstein et al. 2008; Footitt et al. 2011; Footitt et al. 2013; Nakamura et al. 2011). In the wild, seed dormancy is vital for plants to avoid adverse environments, as it ensures that seeds germinate only when environmental conditions are suitable for plant growth and development. It is an adaptive trait and the result of natural selection in wild plants (Finkelstein et al. 2008). During the long history of domestication, seed dormancy has been selectively abandoned to achieve uniform germination and higher germination percentages (Shu et al. 2016; Sugimoto et al. 2010). To reduce PHS, appropriate seed dormancy should be re-introduced.

Many hormones have been reported to regulate seed dormancy, with gibberellins (GAs) and abscisic acid (ABA) being the two most important ones (Footitt et al. 2011; Shu et al. 2016; Vaistij et al. 2013). Transcriptomic analyses of seed development and germination have shown that GAs and ABA play important roles in regulating gene expression for seed dormancy (Sreenivasulu et al. 2008; Wan et al. 2008). During seed development, ABA has a role in suppressing germination. After breaking dormancy, the ABA content decreases and the GAs content increases to promote germination (Shu et al. 2016). The synthesis, degradation, and signal transduction of ABA and GAs play important roles in the conversion between dormancy and germination (Finkelstein et al. 2008; Graeber et al. 2012; Graeber et al. 2014; Holdsworth et al. 2008; Shu et al. 2016)

Most of the dormancy-related genes that have been cloned from cereals so far are involved in the synthesis or signal transduction of GAs or ABA (Jin et al. 2018; Shu et al. 2016). *Sdr4* was the first seed dormancy gene cloned from rice, and its transcription was shown to be controlled by *OsVP1* (a homolog of the ABA-responsive transcription factor ABI3). In *Arabidopsis, AtMFT* was shown to regulate ABA sensitivity and in wheat, *TaMFT* was found to positively regulate seed dormancy (Chono et al. 2015; Li et al. 2014; Nakamura et al. 2011; Tao et al. 2014; Xi et al. 2010). Mutations of *qSD1–2,* which controls GA synthesis, led to decreased GA levels and plant height, and enhanced seed dormancy (Ye et al. 2015a). Although a number of genes involved in seed dormancy have been identified, our knowledge about this process is still limited. To facilitate crop breeding, more details of the mechanisms controlling seed germination need to be dissected.

The MAPK cascade is an evolutionarily conserved signaling module that plays vital roles in eukaryotes, including animals, yeast, and plants. The plant MAPK cascade system is more complex than those of animals because plants cannot escape from harsh environmental conditions. The MAPK cascade converts extracellular stimuli into intracellular signals and plays important roles in various signal transduction pathways (Colcombet and Hirt 2008). Many studies have shown that MAPK cascades mediate the signal transduction of hormones such as ABA, jasmonic acid, and ethylene in response to environmental signals (Choi et al. 2017; Danquah et al. 2015; Li et al. 2017; Matsuoka et al. 2015; Ye et al. 2015b; Yoo et al. 2008). The traditional MAPK cascade system is composed of three kinds of protein kinases: MKKK, MKK, and MAPK. Each kinase in the MAPK cascade module is sequentially activated by a relay of phosphorylation events. The activated MAPK phosphorylates downstream proteins to modify their activities and transmit the corresponding signal. Several entire cascades have been clarified. For example, the MKKK1-MKK2-MAPK4/6 module mediates cold and salt stress tolerance (Teige et al. 2004); and YODA-MKK4/5-MAPK3/6 regulates stomatal development (Lampard et al. 2009). The MKKKs start the cascade, and are activated or transcriptionally regulated by environmental or developmental signals. It is particularly important to understand the functionality of MKKKs. Recently, many reports have shown that the MAPK cascade participates in the regulation of seed germination. The MKK1–MAPK6 module was shown to transmit the ABA signal and control the seed germination process by regulating the expression of *CAT1* and the glucose-induced ABA level (Xing et al. 2008, 2009). Map-based cloning studies have shown that *MKK3* controls seed dormancy in barley and wheat (Nakamura et al. 2016; Torada et al. 2016). Two genes in the Raf subfamily of MKKK genes, *Raf10* and *Raf11,* were found to positively regulate seed dormancy in *Arabidopsis* (Lee et al. 2015).

In this study, we found that the overexpression of *MKKK62* in rice led to the loss of seed dormancy and decreased ABA sensitivity. Protein interaction analyses confirmed interactions between downstream members of the MAPK cascade, and their function in dormancy regulation was verified by knockout experiments. These analyses have revealed the details of the MAPK cascade that controls seed dormancy in rice.

## Results

### Overexpression of *MKKK62* in rice results in loss of seed dormancy

In rice, there are 75 *MKKK*s, eight *MKK*s, and 15 *MAPK*s (Group. M 2002; Hamel et al. 2006; Rao et al. 2010). Relatively few studies have focused on these kinases, especially the MKKKs. It was reported that the expression level of *MKKK62* was greatly affected by the external environment (Rao et al. 2010). To examine the expression pattern of *MKKK62*, we conducted real-time PCR using total RNA extracted from the shoot, leaf, and seed. The transcript level of *MKKK62* was high at the late stage of seed maturation, suggesting that *MKKK62* affected certain characteristics of mature seeds (Additional file 1: Figure S1). To study its function, we cloned *MKKK62* from the cultivar ZH11, a *Japonica* rice variety. The encoded protein was identical to XP_015639022.1. We then overexpressed *MKKK62* in ZH11 under the control of the maize ubiquitin promoter (*Ubi*::*MKKK62*). Three homozygous T_2_ *MKKK62*-overexpressing (OE) transgenic lines (OE1, OE2, OE3) were selected for characterization.

**Fig. 1:**
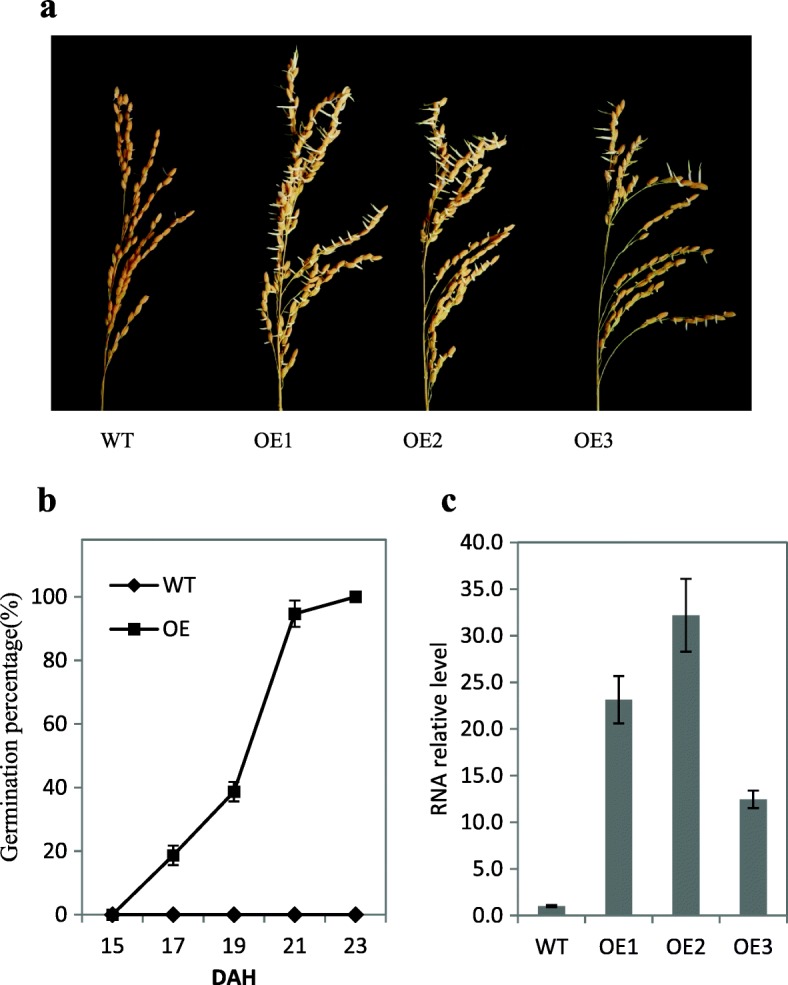
Overexpression of *MKKK62* in ZH11 resulting in PHS. a, Germination phenotype of WT and OE panicles. At 30 DAH, panicles of OE1, OE2, OE3, and WT were sampled for germination tests. Seeds from OE lines germinated at 3 DAI. b, Germination percentage of seeds from OE panicles sampled at indicated times. Seeds were harvested at indicated times from OE plants for germination test. Germination percentage was calculated at 2 DAI. Values represent the mean ± SD of three biological replicates. c, Relative expression level of *MKKK62* in seeds of *MKKK62-*overexpression (OE) lines at 23 DAH. At 23 DAH, gene transcript levels in seeds of OE1, OE2, OE3, and WT were analyzed by real-time PCR

The OEs showed a PHS phenotype. To confirm the phenotype at 30 days after heading (DAH), we collected the panicles and tested the seed germination percentage immediately. At 3 days after imbibition (DAI), all the seeds from homozygous OEs germinated, while few seeds of wild type (WT) germinated (Fig. 1a). To test the germination ability at the seed-filling stage, we harvested seeds at different times, kept them under germination conditions for 2 days, and then calculated the germination percentage. At 2 DAI, several seeds collected from the OEs at 18 DAH germinated, all seeds collected from the OEs at 23 DAH germinated, but none of the WT seeds germinated (Fig. 1b, Additional file 2: Figure S2). The real-time PCR assay results showed that the transcript levels of *MKKK62* were higher in seeds of the three OE lines than in ZH11 seeds at 23 DAH (Fig. 1c).To investigate the effect of *MKKK62* on seed dormancy, we investigated the germination characteristics of seeds from heterozygous OEs. At 23 DAH, we collected the panicles and tested the seed germination percentage. At 2 DAI, the germinated seeds were counted and then planted. Analyses of DNA extracted from the leaves of the seedlings confirmed that all of them contained the marker gene, and so all were transgenic lines. The non-germinating seeds were harvested and cultivated on 1/2 Murashige & Skoog medium, and then analyzed to determine whether they contained the marker gene. None of the plants derived from non-germinated seeds were transgenic lines (Table 1, Additional file 3: Figure S3). This result also confirmed that the overexpression of *MKKK62* led to the loss of seed dormancy.

**Table 1:** Seed germination of progeny of three heterozygous OE lines

	OE1		OE2		OE3	
	+	–	+	–	+	–
Germinated seeds	658	0	564	0	576	0
Non-germinated seeds	0	216	0	180	0	173

Note: Five panicles were harvested at 23 DAH from each of three heterozygous OE lines for the germination test. Numbers of seeds germinated/non-germinated after 2 days are shown. Plus sign, transgenic progeny; minus sign, non-transgenic progeny

### ABA and GAs contents in seeds

**Fig. 2:**
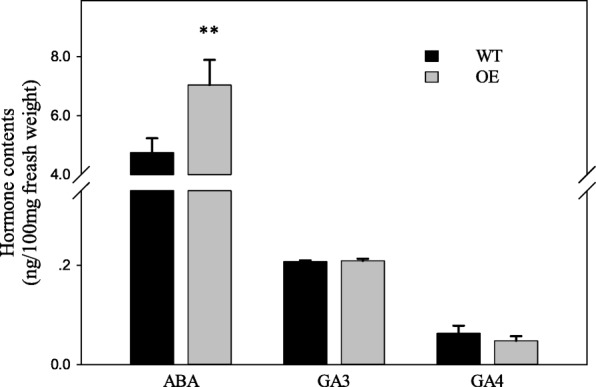
ABA and GAs contents in seeds of OE and WT lines. At 23 DAH, seeds from three OE lines and WT were harvested and stored in liquid nitrogen until analysis. Values represent the mean ± SD of at least three replicates. Student’s t-tests were used to generate *P* values

Both ABA and GAs are important hormones in the regulation of seed dormancy. To determine whether the PHS phenotype of OEs was due to changes in the contents of ABA or GAs, we quantified these hormones in the seeds of WT and the three OEs by LC-MS/MS. The contents of GA_3_ and GA_4_ were similar in the OEs and WT, while the ABA content was significantly higher in the OEs than in WT (Fig. 2).

### Decrease in ABA sensitivity during germination of OE seeds

**Fig. 3:**
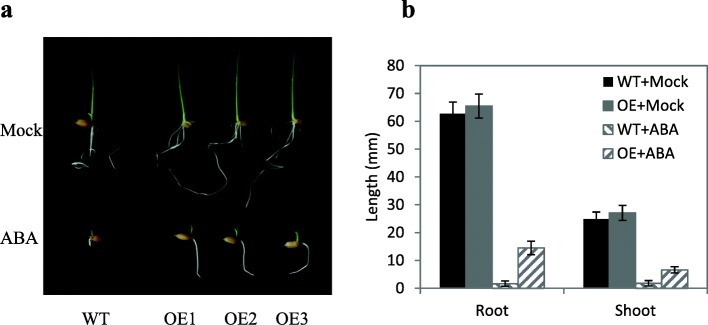
Decreased ABA sensitivity in OE lines. a, Growth performance of OE lines and WT on 1%(*w*/*v*) agar without or with 10 μM ABA. In mock treatment, agar contained equal amount of solvent. b, Length of shoot and root of OE lines after 3 days of treatment. Shoot and root lengths were measured after 3 days of culture in the light at 30 °C.Values represent the mean ± SD of three OE lines (ten replicates/line)

In plants, ABA positively regulates dormancy. To evaluate the effect of ABA to suppress the germination of OE seeds, we treated OE seeds with different concentrations of ABA at 23 DAH. According to this result, 10 μM ABA was suitable to show the suppression effect of ABA on seed germination of OE (Additional file 4: Figure S4). Germination and especially post-germination growth are usually inhibited by ABA (Lopez-Molina et al. 2001). To evaluate the sensitivity of post-germination growth to ABA, identically sprouted seeds of OE and WT were selected and placed on agar containing ABA. After 3 days, the shoots of OE were significantly longer than those of WT in the mock treatment (*P* < 0.05) (Fig. 3a, and b). In the ABA treatment, the shoots and roots of the OE lines were significantly longer than those of WT (*P* < 0.01). Compared with the mock treatment, relative root and shoot elongation was much greater in OE than in WT (Fig. 3a, and b). This result indicated that ABA inhibited shoot and root growth much more strongly in WT than in OE. Together, these results showed that ABA sensitivity was decreased in the OE lines.

### Identification of protein interactions

**Fig. 4:**
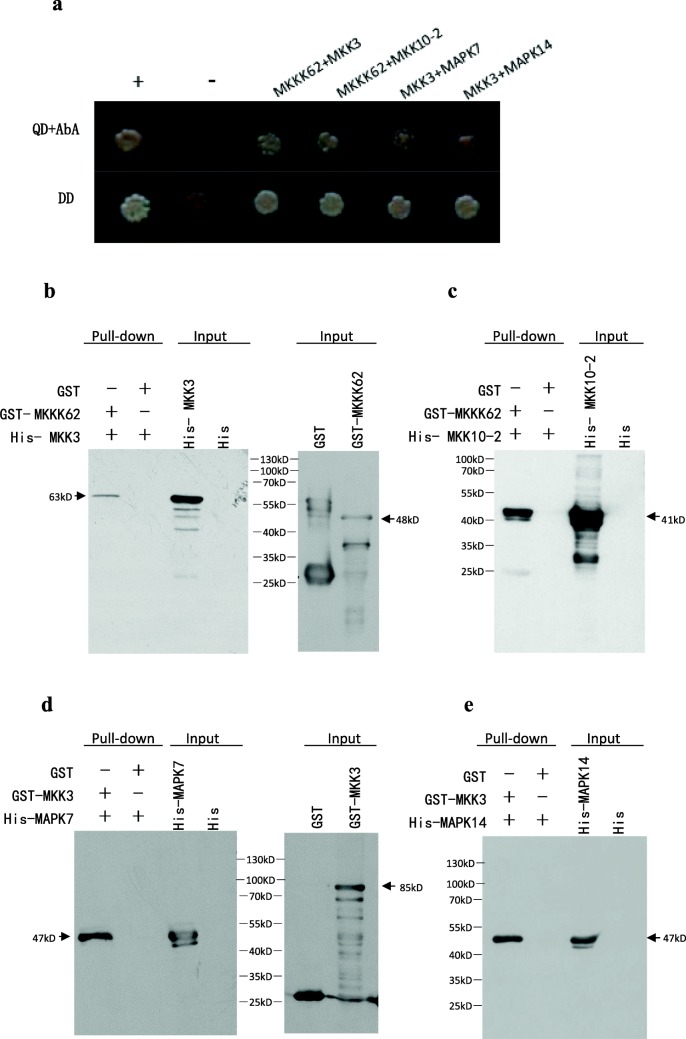
Interactions between indicated proteins. a, Interactions between indicated proteins were investigated by Y2H. Plus sign, positive control; minus sign, negative control; QD + AbA, quadruple-dropout medium lacking Ade, His, Leu and Trp containing 200 ng/mL AbA; DD, double-dropout medium lacking Leu and Trp. b, c, d, and e, pull-down results of four pairs of proteins: MKKK62-MKK3, MKKK62-MKK10–2, MKK3-MAPK7, and MKK3-MAPK14, respectively. Binding of GST- and His-fusion proteins was tested by immunoblotting using anti-His after pull-down with GST-fusion protein. GST was used as negative control. Expression of fusion proteins was detected using antibody to the tag. “Input” indicated the detection result of the fusion proteins with anti-His or anti-GST antibody; “Pull-down” indicated the detection result of His-fusion protein binding to GST-fusion protein with anti-His antibody. Arrow indicates the band of the target protein

The MAPK module usually consists of MKKK-MKK-MAPK. Usually, MKKK62 will interact with and activate downstream MKKs, and the activated MKKs will interact with and activate the downstream MAPKs. To explore these relationships, we performed Y2H experiments. The coding region of *MKKK62* was cloned and inserted into a bait vector (pGBKT7). We amplified the eight rice *MKK* genes using seed cDNA as the template and the primer pairs listed in Additional file 8: Table S2. Only seven MKKs were successfully amplified and cloned into the prey vector (pGADT7). No autoactivation activity was detected in yeast (data not shown). Every MKKK62–MKK pair was co-transformed into yeast cells, and the obtained colonies were cultured on double-dropout medium. The co-transformed cells were then streaked onto quadruple-dropout medium containing 200 ng/mL Aureobasidin A (AbA). According to the growth on quadruple-dropout medium, MKKK62 interacted with MKK3 and MKK10–2. We used a similar method to screen for proteins interacting with MKK3. We found that MKK3 interacted with two of the 15 MAPK proteins, MAPK7 and MAPK14 (Fig. 4a). None of the MAPK proteins interacted with MKK10–2. To further test the interaction, we performed pull-down experiments. The pull-down assay using *E. coli*-produced recombinant proteins confirmed the following interactions: MKKK62-MKK3, MKKK62-MKK10–2, MKK3-MAPK7, and MKK3-MAPK14(Fig. 4b, c, d, and e). Based on the Y2H and pull-down results, MKK3, MKK10–2, MAPK7, and MAPK14 were identified as candidate downstream proteins of MKKK62.

### Knockout of downstream genes increased seed dormancy of OE lines

To explore the roles of downstream proteins of MKKK62 in regulating dormancy, we constructed CRISPER vectors to knockout certain downstream genes. The amino acid sequences of MAPK7 and MAPK14 were similar, suggesting that they may have redundant functions. Therefore, we constructed the vector MB-MAPK7/14 to knock out both genes simultaneously. After *Agrobacterium*-mediated genetic transformation, we obtained 10 independent transgenic lines containing MB-MKK3 and three independent lines containing MB-MAPK7/14.

Using DNA from leaves of T_2_-generation plants as the template, a fragment containing the target site was amplified and sequenced. Progenies with frameshift mutations were selected for dormancy analysis. Tables 2 and 3 show the target sites and sequences of *MKK3, MAPK7,* and *MAPK14* in various knockout progenies.

**Table 2:**
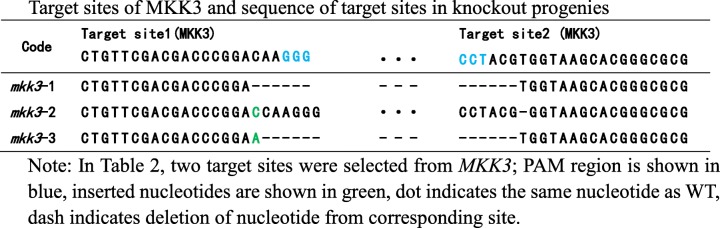
Target sites of MKK3 and sequence of target sites in knockout progenies

**Table 3:**
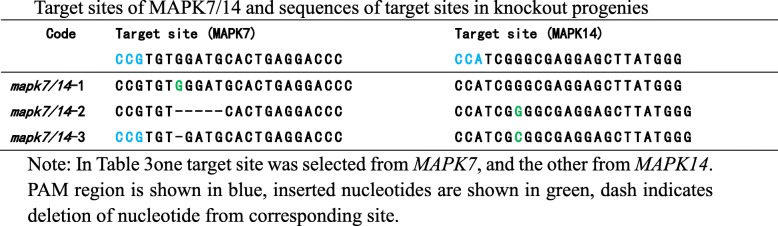
Target sites of MAPK7/14 and sequences of target sites in knockout progenies

**Fig. 5:**
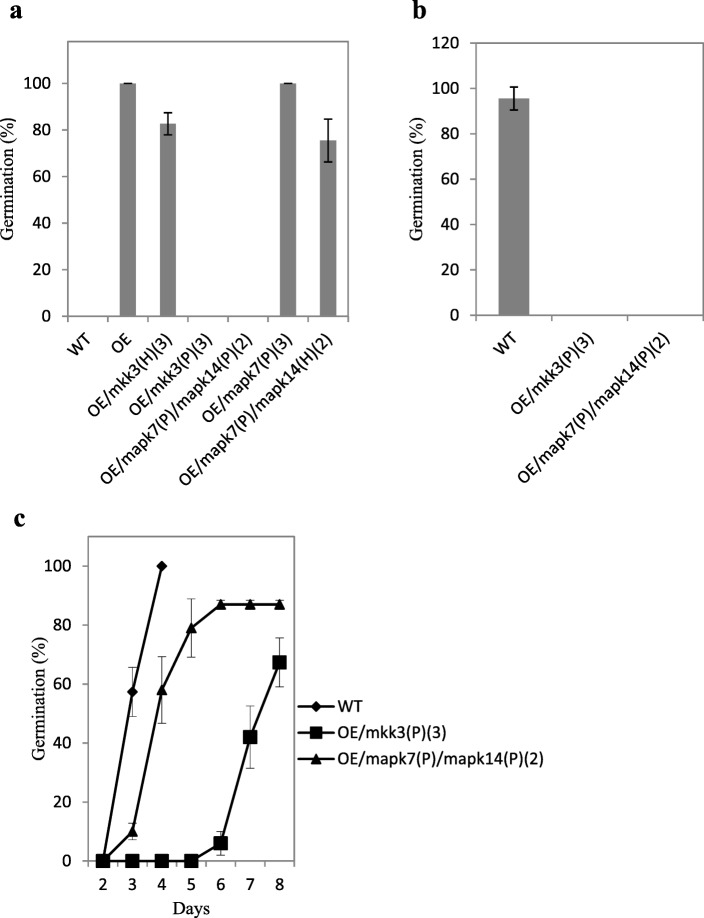
Germination phenotype of knockout progenies. a, At 23 DAH, seeds were collected from different knockout progenies and germination percentage was calculated at 2 DAI. b, At 30 DAH, seeds were collected from homozygous knockout progenies and germination percentage was calculated at 5 DAI. c, Time course of germination of homozygous OE/*mkk3* and OE/*mapk7/mapk14* seeds after storage at 37 °C for 1 month. mkk3, *MKK3* knockout plant; mapk7, *MAPK7* knockout plant; mapk14, *MAPK14* knockout plant; P, homozygous positive; H, heterozygous; Number in parentheses indicates number of knockout lines. Values are mean ± SD

In the late cropping season in 2017, we selected the frame-shift mutants in the T_2_ generation for seed dormancy analysis. Seeds were harvested at 23 DAH for the germination test, and the germination percentage was calculated at 2 DAI. The seeds from homozygous plants of OE/*mkk3* and OE/*mapk7*/*mapk14* did not germinate, but most seeds from heterozygous plants of OE/*mkk3*(H) and OE/*mapk7*(P)/*mapk14*(H) germinated, and all seeds from homozygous plants of OE/*mapk7* germinated (Fig. 5a). Seeds harvested from homozygous plants of OE/*mkk3* and OE/*mapk7*/*mapk14* at 30 DAH did not germinate, even at 5 DAI (Fig. 5b). After 1 month of storage at 37 °C to break dormancy, we tested the germination percentage of seeds from homozygous plants of OE/*mkk3* and OE/*mapk7*/*mapk14*. After breaking dormancy the germination percentage of OE/mapk7(P)/mapk14(P) increased greatly and above 60% of seeds from OE/mkk3(P) also germinated at 8 DAI (Fig. 5c). It indicated that the knockout of MKK3 or MAPK7/14 is nonlethal to seeds. These results revealed that the mutation of *MKK3* or *MAPK7*/*14*/could stop the PHS phenotype induced by the overexpression of MKKK62.

### Expression analysis of genes related to dormancy

**Fig. 6:**
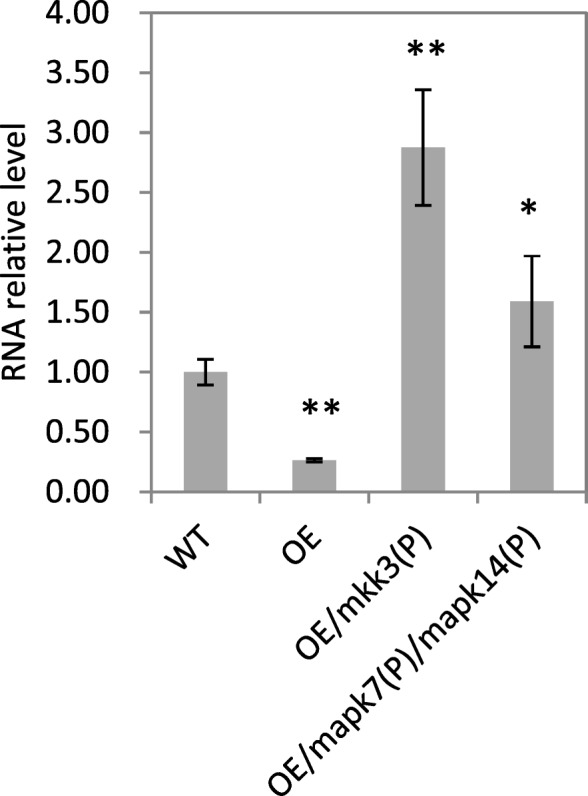
Relative transcript levels of *OsMFT* in different transgenic materials. Transcript level of *OsMFT* was analyzed by real-time PCR using seeds collected at 23 DAH. Values shown are the mean ± SD of triplicate independent biological samples (*n* = 3). Transcript level was normalized to that of *OsMFT* in WT. Student’s *t*-tests were used to generate *P* values

Genes that have been reported to regulate seed dormancy in cereals include *Sdr4*, *OsDOG1-likeOsVp1*, *TaMFT,* and *qSD7–1* (Bentsink et al. 2006; Gu et al. 2011; Hattori et al. 1994; Nakamura et al. 2011; Sugimoto et al. 2010)*.* To investigate whether *MKKK62* affects the expression of these genes, we collected seeds from the OE lines at 23 DAH and analyzed gene transcript levels by real-time PCR. The transcript level of *OsMFT* in seeds was significantly lower in the OE lines than in WT (Fig. 6). There was no significant difference in the transcript levels of *Sdr4*, *OsDOG1-like, OsVp1*, and *qSD7–1* between WT and OE (data not shown). Further analyses showed that the transcript levels of *OsMFT* were much higher in homozygous OE/*mkk3*(P) and OE/*mapk7*(P)/*mapk14*(P) seeds than in WT seeds (Fig. 6). These results indicated that the MAPK cascade controls the expression of *OsMFT* in rice seeds.

## Discussion

With the increasing frequency of climatic anomalies, high temperatures and long-term rain often occur during the rice-harvesting period, especially for early rice crops in tropical and subtropical regions. Therefore, to increase rice production, it is very important to enhance seed dormancy and avoid yield losses from PHS.

### *MKKK62* control seed dormancy

Rice *MKKK*s can be classified into three main subfamilies; the *MEKK, ZIK,* and *Raf* subfamilies. The MEKK subfamily is most similar to animal MEKKs, which phosphorylate and activate the MEK protein. The MEKK subfamily in rice has 22 members, including *MKKK62* (Rao et al. 2010). We detected high transcript levels of *MKKK62* at the late stage of seed maturation, suggesting that *MKKK62* affected seed traits (Additional file 1: Figure S1). Subsequent experiments showed that overexpression of *MKKK62* in ZH11 resulted in the loss of seed dormancy (Fig. 1a, b, Additional file 2: Figure S2). The seed dormancy of N22 (an *Indica* rice variety) is very strong (Wan et al. 2006), but overexpression of *MKKK62* in N22 led to the loss of seed dormancy (Additional file 5: Figure S5). We also overexpressed *LOC103652526* (the maize homolog of *MKKK62*) in rice (ZH11) and the transgenic progeny showed a similar phenotype to that of the OE lines (data not shown). These results indicated that regulation of seed dormancy by *MKKK62* is a common phenomenon among cereals.

Seed dormancy is determined by the seed embryo, endosperm, testa, and husk (Finch-Savage and Leubner-Metzger 2006; Ye et al. 2015a). The genotypes of testa and husk are identical to that of the mother plant, and the embryo genotype was the same as the genotype of the seedling grown from each seed. Endosperm genotype is closely related to embryo genotype. Statistical analyses of seed dormancy in the progenies of heterozygous OE lines showed that all the plants that grew from germinating seeds were transgenic positive and those that grew from dormant seeds were transgenic negative. Thus, the overexpression of *MKKK62* in the embryo and endosperm significantly decreased seed dormancy levels, while the overexpression of *MKKK62* in the testa and husk did not (Table 1, Additional file 3: Figure S3).

We also knocked out *MKKK62* in ZH11 by gene editing technology and obtained three independent lines (Additional file 6: Table S1). However, the germination efficiency (percentage of germinating seeds) of the knockout lines was not obviously decreased (Additional file 7: Figure S6). Our explanation for this result is as follows: In *Arabidopsis*, at least five MKKKs (AtMKKK14/15/16/17/18) are able to activate AtMKK3 (Choi et al. 2017; Matsuoka et al. 2015). In rice, there are at least eight genes showing strong similarity to *MKKK62* (Rao et al. 2010), and six of them are transcribed in seeds (Sato et al. 2013). We speculated that these six genes can functionally complement the mutation of MKKK62. *AtMKKK14*/*15*/*16*/*17*/*18* in *Arabidopsis* also belong to the MEKK subfamily. According to the phylogenetic tree, their predicted amino acid sequences show strong similarity to that of MKKK62 (Rao et al. 2010). However, there were no significant changes in the seed dormancy of *Arabidopsis* overexpressing *AtMKKK14*/*15*/*16*/*17*/*18* (Choi et al. 2017; Matsuoka et al. 2015). In *Arabidopsis,* ABA was shown to positively regulate the expression of *AtMKKK18* and promote the phosphorylation of the downstream proteins, AtMAPK1/2/7/14 (Danquah et al. 2015; Leonhardt et al. 2004). Overexpression of *AtMKKK18* led to accelerated leaf senescence, a phenotype usually induced by ABA (Matsuoka et al. 2015). Seed dormancy is usually promoted by ABA (Jin et al. 2018). Since *AtMKKK18* has been shown to positively regulate the ABA signal (de Zelicourt et al. 2016), it has been speculated that *AtMKKK18* also positively regulates seed dormancy. The results of the present study showed that rice seed dormancy is negatively regulated by *MKKK62*. This result suggests that the regulatory mechanisms of seed dormancy differ between *Arabidopsis thaliana* and rice.

In *Arabidopsis*, *Raf10* and *Raf11* belong to the RAF subfamily of MKKK genes. Freshly harvested seeds of the mutants *raf10* and *raf11* without stratification were able to germinate on MS medium. The seed germination rate of the *raf10/raf11* double mutant was higher than those of the single mutants, indicating that both *Raf10* and *Raf11* are required for seed dormancy (Lee et al. 2015). In rice, there are many homologous genes to *AtMKKK14*/*15*/*16*/*17*/*18*, *Raf11* and *Raf10*. The functions of these genes are still unknown. We believe that further research will reveal more and more of the MKKKs that are involved in the regulation of seed dormancy.

### MKKK62-MKK3-MAPK7/14 module regulates seed dormancy

None of the studies in which *AtMKKK14*/*15*/*16*/*17*/*18* were overexpressed in *Arabidopsis* reported effects on seed dormancy (Choi et al. 2017; Matsuoka et al. 2015), while the overexpression *MKKK62* was found to cause serious PHS in the present study. It is unlikely that *AtMKKK18* and *MKKK62* have similar functions in the regulation of seed dormancy. In addition, the nomenclature of MAPK proteins is inconsistent, and many protein interactions in MAPK cascade in *Arabidopsis* need to be re-validated in rice.

To further explore the regulatory mechanism of MKKK62, we used Y2H experiments to screen for interactions between MKKs proteins and MKKK62 using MKKK62 as the bait. MKK3 and MKK10–2 were screened from seven MKK proteins. In *Arabidopsis*, similar proteins (AtMKKK14/15/16/17/18) were found to interact with AtMKK3 in Y2H, bimolecular fluorescence complementation, and pull-down analyses (Choi et al. 2017). AtMKKK14/15/16/17/18 were also shown to interact with AtMKK2/4/5, although the interactions were weaker. We did not observe similar results in the present study.

In this study, MAPK7 and MAPK14 interacted with MKK3 (Fig. 4a, d, and e). According to the phylogenetic relationships of *Arabidopsis* and rice MAPK genes, MAPK7 and MAPK14 belong to Clade C in the MAPK phylogeny (Hamel et al. 2006). In a previous study, all members of C clade were found to interact with MKK3 in *Arabidopsis* (Dóczi et al. 2007). The results of our study were consistent with this conclusion. In other study, MKK10–2 was found to interact with MAPK7 (Wankhede et al. 2013), but we did not observe this interaction in our study. It is possible that our screening conditions were more stringent (200 ng/mL AbA in quadruple-dropout medium), and so weak interactions were not detected.

It has been reported that MKK3 can conduct the jasmonic acid signal by activating MAPK6(Sethi et al. 2014; Takahashi et al. 2007). We did not detect any interaction between MKK3 and MAPK6 in our Y2H experiments, suggesting that MAPK6 may be activated indirectly by MKK3. The interaction between MKKK62 and MKK4 predicted in a previous study (Jung et al. 2010) was not detected in our study. These results reflect the complexity of the MAPK signaling system, and highlight the importance of experimentally verifying all interactions in the possible MAPK cascade. At 23 DAH, compared with OE, OE/*mkk3*(P) and OE/*mapk7*(P)/*mapk14*(P) re-obtained seed dormancy (Fig. 5a). After maturity, seed dormancy was stronger in OE/*mkk3*(P) and OE/*mapk7*(P)/*mapk14*(P) than in WT (Fig. 5b, and c). It indicated that *MKK3*, *MAPK7*, and *MAPK14* participate in dormancy regulation and function downstream of MKKK62. Combined with the Y2H and pull-down results, these findings implied that the MKKK62-MKK3-MAPK7/14 module regulates seed dormancy and that MKK10–2does not belong to this MAPK module. During the course of our research, *MKK3* was found to control seed dormancy in wheat and barley using map-based cloning method (Nakamura et al. 2016; Torada et al. 2016). In this paper, we described in detail each number of the MAPK module that controls seed dormancy in rice.

### Decreased ABA sensitivity in OE results in the loss of seed dormancy

The main hormones controlling seed dormancy are ABA and GAs. In *Arabidopsis,* ABA was shown to increase the expression level of *AtMKKK18* (Matsuoka et al. 2015). In this study, the ABA content was higher in OE than in WT (Fig. 2). These results indicate that the MAPK cascade not only responds to the ABA signal, but also influences the ABA level. The OE plants in this study lost seed dormancy. We speculated that the ABA sensitivity of the OE lines was decreased. The ABA treatment inhibited the shoot and root growth of OE and WT, but the inhibition was greater in WT than in OE (Fig. 3a, and b). This result was consistent with our speculation that OE lines had decreased ABA sensitivity. In *Arabidopsis*, the transcription of *AtMFT* was promoted by ABA (Xi et al. 2010). In our study, the OE lines showed increased ABA content, but decreased transcript levels of *OsMFT* (Fig. 6), indicating that ABA signal transduction was inhibited in the OE lines. These results also supported that ABA sensitivity was reduced in the OE lines.

The *fy-1* mutant in *Arabidopsis* germinated quickly without stratification or after-ripening, Further analyses showed that the ABA sensitivity of *fy-1* was lower than that of WT. However, the ABA level was higher in *fy-1* seeds than in WT seeds, despite the stronger dormancy of WT seeds than *fy-1* seeds (Jiang et al. 2012). A previous study reported that the *Arabidopsis* mutants *raf10* and *raf11* were less sensitive to ABA inhibition of seed germination than was WT, and the overexpression of *Raf10* resulted in delayed seed germination and enhanced ABA sensitivity (Lee et al. 2015). All these results suggested that ABA sensitivity is more important than ABA content in germination progress. When seeds of *AtMKKK16*-OE lines and WT were treated with ABA, the relative germination percentages were higher in the *AtMKKK16*-OE lines than in WT. However, primary root elongation in the *AtMKKK16*-OE lines was found to be slightly hypersensitive to ABA inhibition (Choi et al. 2017). These results indicated that the ABA sensitivity of the primary root was not decreased in *AtMKKK16*-OE lines, which would explain why the seed dormancy was not greatly affected in *AtMKKK16*-OE.

### MKKK62-MKK3-MAPK7/MAPK14 regulates *OsMFT* to mediate seed dormancy

**Fig. 7:**
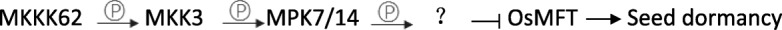
Model of regulation of seed dormancy in rice. Arrows indicate transcriptional activation, flat-ended arrows indicate transcriptional repression. Arrows with ℗ indicate activation by phosphorylation. Question mark indicates unknown part

*MFT* encodes a protein in the phosphatidyl ethanolamine-binding protein family. In wheat, the down-regulation of *TaMFT* under high temperature led to PHS, and the overexpression of *TaMFT* suppressed germination(Nakamura et al. 2011). The mis-splicing of *TaMFT* was shown to cause PHS susceptibility (Liu et al. 2015).The results of those studies showed that the transcriptional level of *TaMFT* controlled seed dormancy. In *Arabidopsis* and soybean, MFT proteins were also found to promote seed dormancy (Dave et al. 2016; Li et al. 2014; Vaistij et al. 2013). In the present study, the transcript levels of *OsMFT* were significantly decreased in OE and significantly increased in OE/*mkk3*(P) and OE/*mapk7*(P)/*mapk14*(P) (Fig. 6). Seed dormancy was also decreased in OE and increased in OE/*mkk3*(P) and OE/*mapk7*(P)/*mapk14*(P) (Fig. 1a, and Fig. 5a, b). Thus, we speculated that the MKKK62-MKK3-MAPK7/MAPK14 module controls seed dormancy by regulating the transcription of *OsMFT* (Fig. 7).

## Conclusion

The overexpression of *MKKK62* in rice decreased seed dormancy levels and ABA sensitivity at the germination stage. We screened the downstream proteins of MKKK62 by Y2H and pull-down analyses and clarified their function in dormancy regulation by knockout experiment. These results revealed the entire MAPK module, MKKK62-MKK3-MAPK7/14, that controls seed dormancy in rice. Expression analyses showed that the MKKK62-MKK3-MAPK7/14 module may control seed dormancy by regulating the expression of *OsMFT*. Further research is required to understand how *OsMFT* is regulated by MKKK62-MKK3-MAPK7/14 and what relationships exist among ABA, GAs, and the MKKK62-MKK3-MAPK7/14 module.

### Methods

#### Plant materials and growth conditions

The rice cultivar ZH11 and N22 were used in this study. ZH11 is a *Japornica* rice variety with weak seed dormancy, and N22 is an *Indica* rice variety with strong seed dormancy(Wan et al. 2005). All materials were grown in the greenhouse of Guangdong Academy of Agricultural Sciences in Guangdong, China.

#### Germination test

The panicles or at least 30 rice seeds were dipped in water and then placed in an incubator (30 °C, humidity > 95%). The germination percentage was calculated at indicated times.

#### Development of *MKKK62*-overexpression plants

The *MKKK62* coding sequence (CDS) was amplified using the primers OX-F and OX-R from cDNA from seeds of the rice cultivar ZH11 (all primers used in this study are listed in Additional file 8: Table S2). The PCR product was double-digested with *Hind*III/*Bam*HI and cloned into the pOX vector to place *MKKK62* under the control of the ubiquitin promoter. Hygromycin B phosphotransferase was the selectable marker. The vector was electroporated into *Agrobacterium tumefaciens* EHA105, and then introduced into the rice varieties ZH11 and N22 via *Agrobacterium*-mediated genetic transformation.

#### Y2H assays

The Matchmaker® Gold Yeast Two-Hybrid System (Clontech, Palo Alto, CA, USA) was used to evaluate protein–protein interactions. We used cDNA obtained from ZH11 as the template for PCR amplification of *MKKK62, OsMAPKs,* and *OsMKKs*, with specific primers containing restriction enzyme recognition sequences. The bait gene was inserted into the pGBKT7 vector, the prey gene was inserted into the pGADT7 vector and the autoactivation activity was tested. These clones were used for Y2H screening. For yeast transformation, yeast competent cells (Y2HGold) were prepared according to the manufacturer’s instructions (PT4084–1, Clontech). Possible interacting constructs were co-transformed into Y2HGold competent cells, and co-transformants were initially screened on double-dropout medium lacking Leu and Trp (SD/−Leu/−Trp). The co-transformed cells were then streaked onto quadruple-dropout medium deficient in Ade, His, Leu, and Trp (SD/−Ade/−His/−Leu/−Trp). The genes used in these analyses were as follows: rice *MKKK62* (*LOC_Os01g50420*), seven rice *MKKs (*(*LOC_Os06g27890* (*OsMKK3*), *LOC_Os03g12390* (*OsMKK10–2*), *LOC_Os06g05520* (*OsMKK1*), *LOC_Os01g32660* (*OsMKK6*), *LOC_Os02g54600* (*OsMKK4*), *LOC_Os06g09180* (*OsMKK5*), and *LOC_Os02g46760* (*OsMKK10–1*)), and 15 rice *MAPKs (*(*LOC_Os02g05480* (*OsMAPK14*), *LOC_Os06g48590* (*OsMAPK7*), *LOC_Os03g17700* (*OsMAPK3*), *LOC_Os10g38950* (*OsMAPK4*), *LOC_Os06g06090* (*OsMAPK6*), *LOC_Os11g17080* (*OsMAPK16*), *LOC_Os06g49430* (*OsMAPK17–1*), *LOC_Os02g04230* (*OsMAPK17–2*), *LOC_Os01g43910* (*OsMAPK20–1*), *LOC_Os05g50560* (*OsMAPK20–2*), *LOC_Os06g26340* (*OsMAPK20–3*), *LOC_Os01g47530* (*OsMAPK20–4*), *LOC_Os05g49140* (*OsMAPK20–5*), *LOC_Os05g50120* (*OsMAPK21–1*), and *LOC_Os01g45620* (*OsMAPK21–2*)).

#### GST pull-down assay

*MKKK62* and *MKK3* fragments were cloned into pGEX-4 T-1 in frame with the GST tag to generate GST-MKKK62 and GST-MKK3 constructs. *MKK3*, *MKK10–2*, *MAPK7,* and *MAPK14* fragments were cloned into the pET-28a(+) vector in frame with the His tag to generate His-MKK3, His-MKK10–2, His-MAPK7, and His-MAPK14 constructs. Details of primers are given in Additional file 8: Table S2. All the constructed vectors were transformed and expressed in *E. coli* strain BL21. GST-fused proteins were extracted and incubated with Glutathione Sepharose™ 4B beads at 4 °C for 1 h. The beads were collected by centrifugation and washed three times with lysis buffer. His-fused proteins were added and incubated at 4 °C overnight. The beads were collected and washed three times with lysis buffer. Finally, the proteins were eluted with reduced glutathione, boiled with loading buffer, and analyzed on a 10% SDS-PAGE gel. Diluted anti-GST antibody and anti-His antibody were used for immunoblotting analysis.

#### Development of knockout lines

Target sites were selected for each gene (*MKK3*, *MKK20–1*, *MAPK7*, *MAPK14*) using tools at the following URL: http://cbi.hzau.edu.cn/crispr/. Two target sites for MKK3 were selected. According to the literature (Ma et al. 2015), the corresponding primers were synthesized and expression cassettes were obtained by overlapping PCR. The expression cassettes were respectively inserted into pYLCRISPR/Cas9Pubi-B to construct the knockout vector MB-MKK3. The knockout vector MB-MKK20–1 was constructed using a similar method. Two target sites (one for *MAPK7* and the other for *MAPK14*) were selected to construct the knockout vector MB-MAPK7/14. Phosphinothricin acetyltransferase was the selectable marker. The vectors were introduced into OE1 via *Agrobacterium*-mediated genetic transformation. To analyze the transgenic lines, we designed detection primers for each gene to amplify the fragment containing the target sites. The amplification products were sequenced. According to the sequencing results, knockout lines were screened for subsequent experiments.

#### Measurement of ABA and GAs contents

The ABA and GAs were extracted from rice seeds as described elsewhere (Pan et al. 2010), and analyzed by liquid chromatography - mass spectrometry (LC-MS/MS). For these analyses, we used a reversed phase ultra-fast liquid chromatograph (Shimadzu, Kyoto, Japan) coupled with a tandem triple quadrupole mass spectrometer (API4000, AB SCIEX, Foster City, CA, USA). The hormone compounds were quantified in multiple reaction monitoring (MRM) mode using the optimized MS/MS conditions listed in Additional file 9: Table S3. The MS conditions were as follows: source, turbo ion spray; ion polarity, negative; ion spray voltage, − 4500 V; source temperature, 550 °C; gas, nitrogen; curtain gas, 30 psi; nebulizing gas (GS1), 55 psi; Collision gas (GS2), 55 psi; scan type, MRM; Q1 resolution: unit; Q3 resolution: unit. Analyst 1.5.2 software (AB SCIEX) was used to control the instrument and to acquire and process all MS data.

#### Gene transcript analyses by real-time PCR

To compare the transcriptional levels of selected genes between transgenic and WT plants, seeds or leaves were sampled from three biological replicates at indicated times and immediately frozen in liquid nitrogen. We extracted RNA from samples (each 300 mg) using Trizol, and 1 μg total RNA was reverse-transcribed into cDNAs. Real-time PCR primers were designed based on the Nipponbare genome sequence (Kawahara et al. 2013). The primers and SYBR Premix ExTaq (Takara, Otsu, Japan) were used to amplify the target gene from the cDNA template(Details about the primers are listed in Additional file 8: Table S2). The *EF1α* gene(*LOC_Os03g08020*) was used as the internal control.

## Additional Files

Additional file 1: **Figure S1.** Expression pattern of *MKKK62*. Transcript level of *MKKK62* was analyzed by real-time PCR using total RNA isolated from shoots (Sh), leaves (L), and seeds (S). Number following S indicates number of days after heading when seeds were sampled. Transcript level was normalized to that of *MKKK62* at 30 DAH. Values shown are mean ± SD of three independent biological samples. (DOCX 17 kb)

Additional file 2: **Figure S2.** Germination phenotype of WT and OE lines at 23 DAH. Panicle branches were harvested at 23 DAH and kept under germination conditions for 2 days. (DOCX 885 kb)

Additional file 3: **Figure S3.** PCR detection of hygromycin gene from OE progenies. M, Marker DL2000; lanes 1–10, samples of non-germinated seeds at 23 DAH; lanes 11–20, samples of germinated seeds at 23 DAH. (DOCX 147 kb)

Additional file 4: **Figure S4.** Germination phenotype of seeds harvested at 23 DAH under ABA treatment. a, Germination phenotype of OE lines after 2 days treatment with ABA; b, Effect of ABA concentration on shoot growth of OE lines. OE seeds were harvested at 23 DAH and placed in a 9-cm Petri dish containing filter paper; 10 ml ABA solution with indicated concentration was added. In mock treatment, water containing an equal volume of solvent was added. Values are mean ± SD of three OE lines (ten replicates/line). (DOCX 171 kb)

Additional file 5: **Figure S5.** Overexpression of *MKKK62* decreased seed dormancy of N22. Overexpression vector of MKKK62 was introduced into N22 by *Agrobacterium-*mediated transformation. Three overexpression lines (N22-OE1, N22-OE2, and N22-OE3) were selected for germination test. In T_0_ generation, panicles of OE lines were harvested at 30 DAH for germination tests. At 2 DAI, most OE seeds germinated while seeds of N22 did not. (DOCX 611 kb)

Additional file 6: **Table S1.** Target sites of *MKKK62* and sequence results of MKKK62-knockout lines (DOCX 17 kb)

Additional file 7: **Figure S6.** Time course of germination of *mkkk62* seeds. In the late cropping season in 2017, seeds were harvested at 30 DAH and kept under germination conditions immediately. Germination percentage was scored daily for 5 days. *mkkk62*–1, *mkkk62*–2 and *mkkk62*–3 are three independent MKKK62-knockout lines. Values shown are mean ± SD of three replicates. (DOCX 17 kb)

Additional file 8: **Table S2.** Primers used in this paper. (DOCX 61 kb)

Additional file 9: **Table S3.** Characteristic fragment ions of hormone standards and optimized MS/MS conditions. (DOCX 15 kb)

## Abbreviations

ABA: Abscisic acid
AbA: Aureobasidin A
DAH: Days after heading
DAI: Days after imbibition
MAPK: Mitogen-activated protein kinase
MKK: Mitogen-activated protein kinase kinase
MKKK: Mitogen-activated protein kinase kinase kinase
OE: Overexpression lines
PHS: Pre-harvest sprouting
WT: Wild type
