# Supplementary figures and images for: The MKKK62-MKK3-MAPK7/14 module negatively regulates seed dormancy in rice
Source: Rice (N Y). 2019 Jan 22;12:2. doi: 10.1186/s12284-018-0260-z (PMC6342742; doi:10.1186/s12284-018-0260-z)

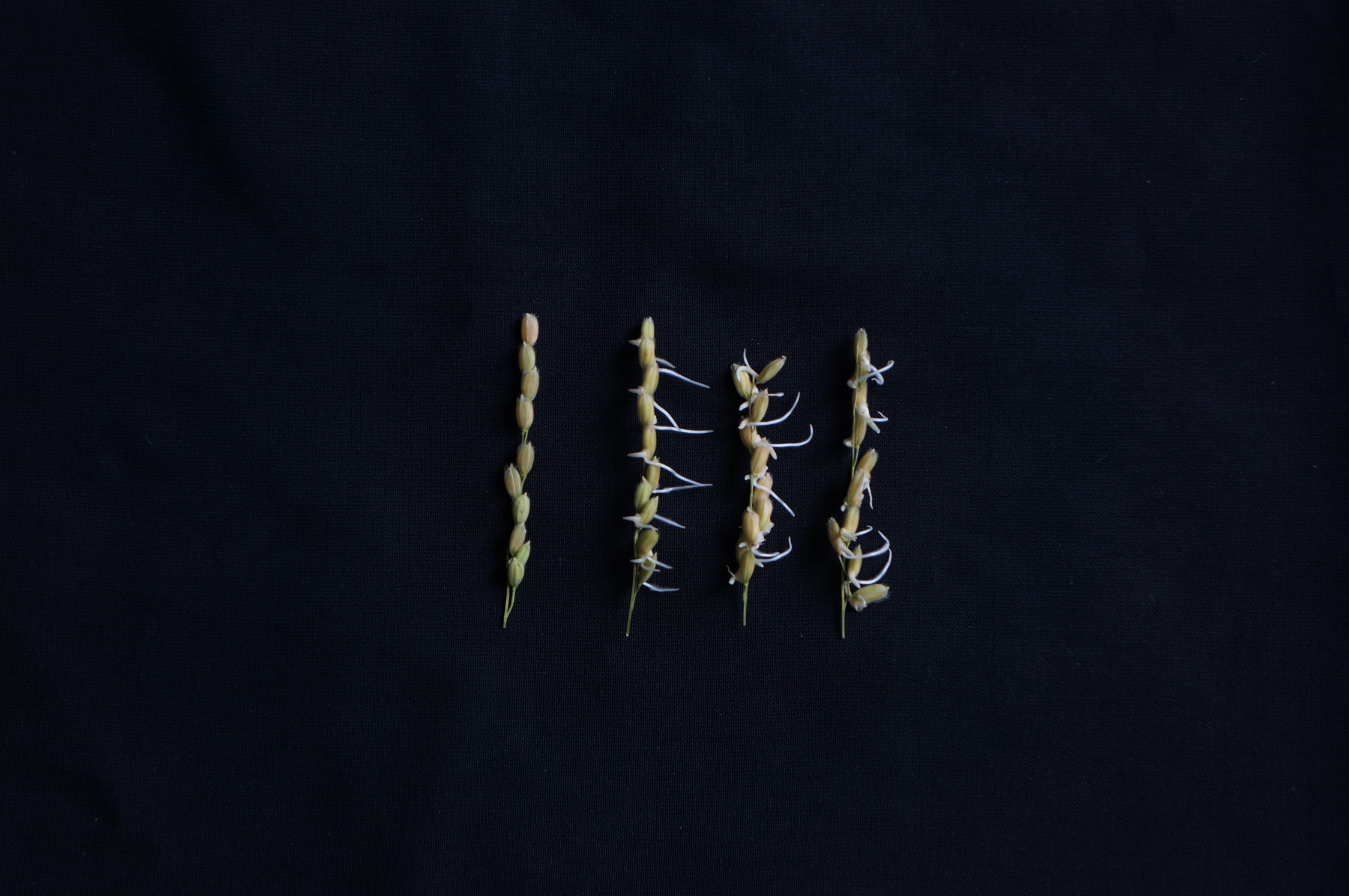


WT OE1 OE2 OE3

Supplement: Supplementary file 2 — Figure S2. Germination phenotype of WT and OE lines at 23 DAH. Panicle branches were harvested at 23 DAH and kept under germination conditions for 2 days. (DOCX 885 kb) [file 12284_2018_260_MOESM2_ESM.docx]

M 1 2 3 4 5 6 7 8 9 10 11 12 13 14 15 16 17 18 19 20


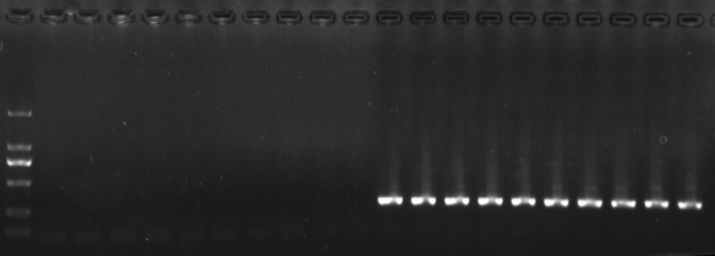

Supplement: Supplementary file 3 — Figure S3. PCR detection of hygromycin gene from OE progenies. M, Marker DL2000; lanes 1–10, samples of non-germinated seeds at 23 DAH; lanes 11–20, samples of germinated seeds at 23 DAH. (DOCX 147 kb) [file 12284_2018_260_MOESM3_ESM.docx]

**a b**


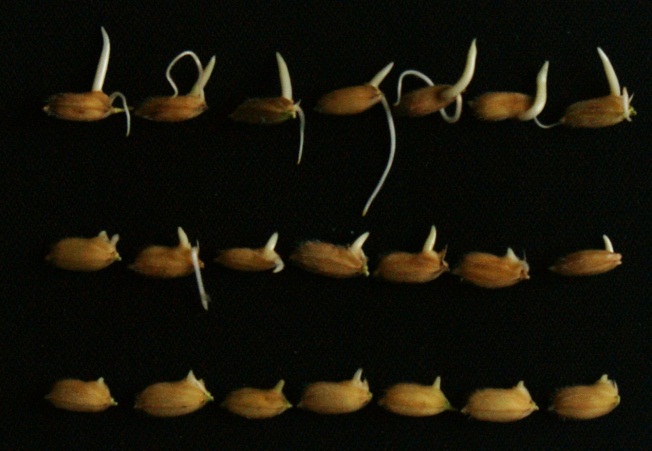


Mock

ABA(10μM)

ABA(100μM)

OE1 OE2 OE3

Supplement: Supplementary file 4 — Figure S4. Germination phenotype of seeds harvested at 23 DAH under ABA treatment. a, Germination phenotype of OE lines after 2 days treatment with ABA; b, Effect of ABA concentration on shoot growth of OE lines. OE seeds were harvested at 23 DAH and placed in a 9-cm Petri dish containing filter paper; 10 ml ABA solution with indicated concentration was added. In mock treatment, water containing an equal volume of solvent was added. Values are mean ± SD of three OE lines (ten replicates/line). (DOCX 171 kb) [file 12284_2018_260_MOESM4_ESM.docx]

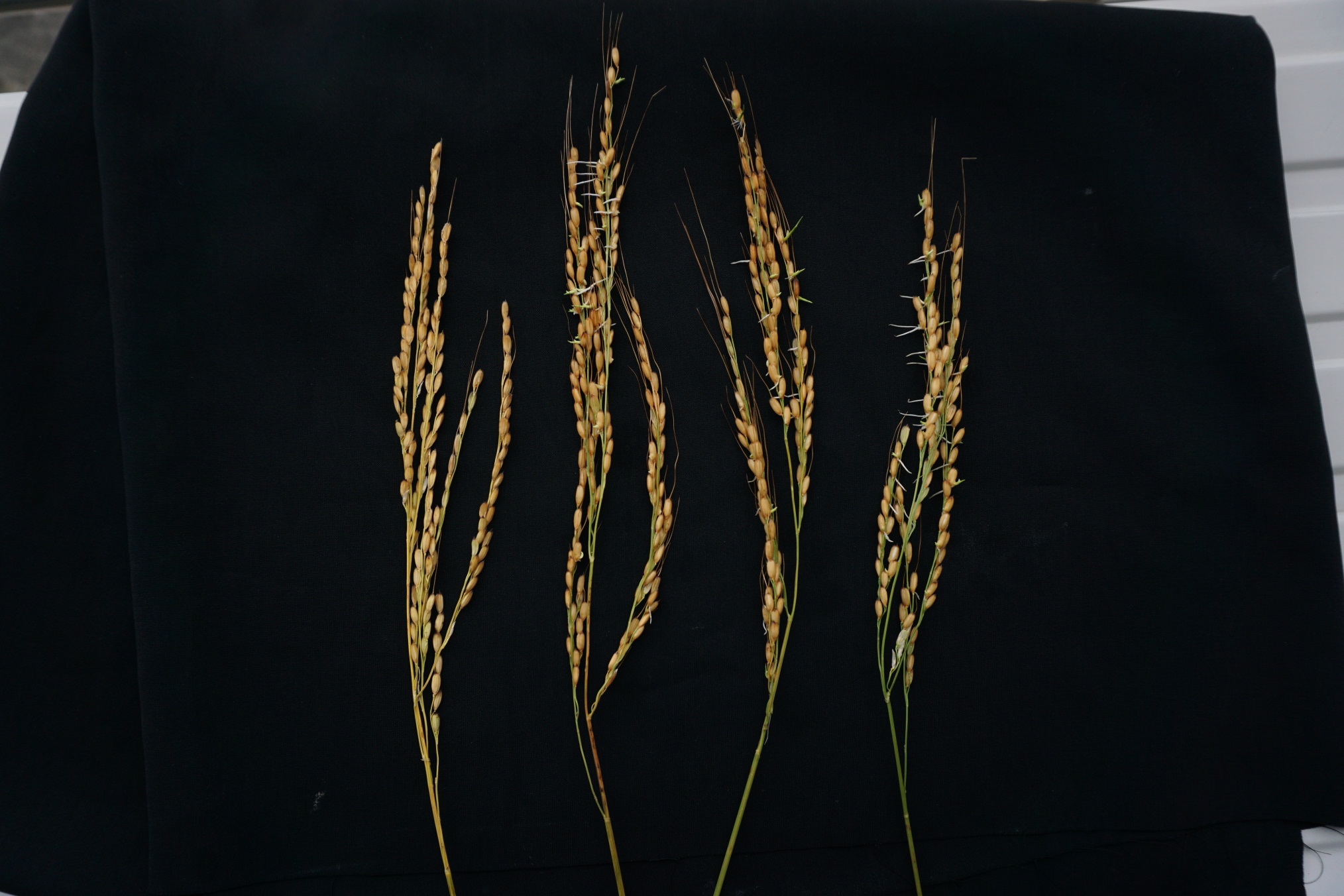


N22 N22-OE1 N22-OE2 N22-OE3

Supplement: Supplementary file 5 — Figure S5. Overexpression of MKKK62 decreased seed dormancy of N22. Overexpression vector of MKKK62 was introduced into N22 by Agrobacterium-mediated transformation. Three overexpression lines (N22-OE1, N22-OE2, and N22-OE3) were selected for germination test. In T0 generation, panicles of OE lines were harvested at 30 DAH for germination tests. At 2 DAI, most OE seeds germinated while seeds of N22 did not. (DOCX 611 kb) [file 12284_2018_260_MOESM5_ESM.docx]
