# Supplementary material for: The MKKK62-MKK3-MAPK7/14 module negatively regulates seed dormancy in rice
Source: Rice (N Y). 2019 Jan 22;12:2. doi: 10.1186/s12284-018-0260-z (PMC6342742; doi:10.1186/s12284-018-0260-z)
Supplement: Supplementary file 7 — Figure S6. Time course of germination of mkkk62 seeds. In the late cropping season in 2017, seeds were harvested at 30 DAH and kept under germination conditions immediately. Germination percentage was scored daily for 5 days. mkkk62–1, mkkk62–2 and mkkk62–3 are three independent MKKK62-knockout lines. Values shown are mean ± SD of three replicates. (DOCX 17 kb) [file 12284_2018_260_MOESM7_ESM.docx]

**Table S1.** Target sites of *MKKK62* and sequence results of MKKK62-knockout lines

| **Code** | **Target site1** |  | **Target site2** |
| --- | --- | --- | --- |
|  | **tgatgcagaagcagctcagg** | **···** | **ggagctgctggcggtcaagtcgg** |
| ***mkkk62*-1** | **tgatgcagaagcagctcAagg** | **···** | **ggagctgctggcggtcaTagtcgg** |
| ***mkkk62-*2** | **tgatgcagaagcagctcAagg** | **···** | **ggagctgctggcggtcaAagtcgg** |
| ***mkkk62-*3** | **tgatgcagaagcagctcTagg** | **···** | **ggagctgctggcggtcaTagtcgg** |

Note: In Table S1, two target sites were selected from *MKKK62*; PAM region is shown in blue, inserted nucleotides are shown in green, dot indicates the same nucleotide as WT.
