# Supplementary material for: The MKKK62-MKK3-MAPK7/14 module negatively regulates seed dormancy in rice
Source: Rice (N Y). 2019 Jan 22;12:2. doi: 10.1186/s12284-018-0260-z (PMC6342742; doi:10.1186/s12284-018-0260-z)
Supplement: Supplementary file 8 — Table S2. Primers used in this paper. (DOCX 61 kb) [file 12284_2018_260_MOESM8_ESM.docx]

Table S2: The primers used in this paper

| Primer Name | | Sequence(5'-->3') |  |
| --- | --- | --- | --- |
| **Transgenic lines** | | |  |
| OX-F | | GGAGAAGCTTCCAAGACCTTCGAAAGCTCG |  |
| OX-R | | GTAGGGATCCTGGTGGAAAGATGTCAACTCGGT |  |
| MKKK62-6aF | | CCAATCGGCCGCTCGGAGTcggcagccaagccag |  |
| MKKK62-6aR | | ACTCCGAGCGGCCGATTGGgttttagagctagaaat |  |
| MKKK62-6bF | | ACTGCCGCGCAGCTACTGGAcaacacaagcggcagc |  |
| MKKK62-6bR | | TCCAGTAGCTGCGCGGCAGTgttttagagctagaaat |  |
| OsMKK3-6aF | | TTGTCCGGGTCGTCGAACAGcggcagccaagccagca |  |
| OsMKK3-6aR | | CTGTTCGACGACCCGGACAAgttttagagctagaaat |  |
| OsMKK3-6bF | | ACGTGGTAAGCACGGGCGCGcaacacaagcggcagc |  |
| OsMKK3-6bR | | CGCGCCCGTGCTTACCACGTgttttagagctagaaat |  |
| OsMKK10-2-6bF | | TCGGTGGCGCGCCTTGTACAcaacacaagcggcagc |  |
| OsMKK10-2-6bR | | TGTACAAGGCGCGCCACCGAgttttagagctagaaat |  |
| OsMPK7-6aF | | TGTGGATGCACTGAGGACCcggcagccaagccagca |  |
| OsMPK7-6aR | | GGTCCTCAGTGCATCCACAgttttagagctagaaat |  |
| OsMPK14-6aF | | TCGGGCGAGGAGCTTATGGGcggcagccaagccagca |  |
| OsMPK14-6aR | | CCCATAAGCTCCTCGCCCGAgttttagagctagaaat |  |
| **Analyze the knockout lines** | | |  |
| MKKK62-decF | | AGCAGCCACCAATACAAATACCT |  |
| MKKK62-decR | | CCTTCTTCTACTTCGTCGTCTTCA |  |
| OsMKK3-decF | | AAGGCTCAGCAAACAAAGGTCA |  |
| OsMKK3-decR | | AGCAGAGGAAGCAGCCAAGC |  |
| OsMPK14-decF | | AACTGTTGGAATCTGGGTTGGA |  |
| OsMPK14-decR | | TGGGATGGGTAACCAAGGGA |  |
| OsMPK7-decF | | GGGTGCCCTTAGGTGTTTCAG |  |
| OsMPK7-decR | | CATGGTGGAACCGCTGCTT |  |
| **Y2H** | |  |  |
| MKKK62E1F | | GGCTTAGAATTCGTGAGATCGATGGATGCGGCT |  |
| MKKK62salR | | GCACGTCGACTTAAAAATTGCTCATTCTAGTCAAAACAGTG |  |
| OsMKK3BIF | | CTAGGGATCCTAATGGCGGGGCTCGAGGAGCTGAAGAAGAA |  |
| OsMKK3salR2 | | CACGGTCGACTCAGGCTTGGATGATGTATAGATCTTGC |  |
| OsMKK10-2E1F | | GACTGAATTCCTCCAACCGATGGCTCTCGT |  |
| OsMKK10-2BIR | | CTCTGGATCCCTATAGATCGCCGCTCTGCTC |  |
| OsMPK7E1F | | GACTGAATTCTTGATGGCGATGATGGTGGAC |  |
| OsMPK7B1R | | CTGAGGATCCGCCTGTCACATATTCACTCCTGC |  |
| OsMPK14E1F | | GACAGAATTCATGGCGATCATGGTGGATCCT |  |
| OsMPK14BglR2 | | GACTAGATCTGATCATCGGGCACTCATTGC |  |
| OsMKK1BIF | | CAACGGATCCTAATGGGGAAGCCGGGGAAGCTA |  |
| OsMKK1SacR | | CTGTGAGCTCTATCTGTCATCATATCGGTTGCCT |  |
| OsMKK6E1F | | GACAGAATTCATGAGGGGGAAGAAGCCGCACAAG |  |
| OsMKK6BglR | | GAGCAGATCTCAAGTTACTCGGATATATTCATTGGAGGT |  |
| OsMKK4ClaF | | GAACTatcgatATATGCGACCGGGCGG |  |
| OsMKK4ClaF | | GAACTatcgatACCAGAAAACGCACACGCC |  |
| OsMKK5E1F2 | | CACTGAATTCATGCGTGCGGGCGACATG |  |
| OsMKK5BglR | | GACAAGATCTGAATTGGCAGTCACGACGATG |  |
| OsMKK10-1E1F | | GACAGAATTCACAATGGCAATGGCGAAGC |  |
| OsMKK10-1BIR | | GTGTGGATCCTATTCTTCTCCGTAGATGCTCGTGT |  |
| OsMPK3E1F | | GTGAGAATTCGAGGAGGGATTAGGGATGGACG |  |
| OsMPK3B1R | | CTGAGGATCCAATCTAGTACCGGATGTTTGGGT |  |
| OsMPK4E1F | | GTGAGAATTCATGGCCATGGATTCCTCCTC |  |
| OsMPK4pstR | | CTGACTGCAGCTGAGCTCTTAGTAGGGAGGATC |  |
| OsMPK6claF | | GACTATCGATACGATCCAAATCCGAATCCG |  |
| OsMPK6B1R | | GACAGGATCCGCTACTGGTAATCAGGGTTGAACG |  |
| OsMPK16ClaF | | GAGCATCGATTCATGGACTTCTTTACCGAGTATGGTG |  |
| OsMPK16BIR | | GACTGGATCCTCAGGGGTAAGAGCCTCGGGGAGAAAC |  |
| OsMPK17-1claF | | CACTATCGATtgGAACTGGTAGGGAGGGGGATG |  |
| OsMPK17-1B1R | | GACTGGATCCGTCTAGGAGTGCATCCTGGAGACTTG |  |
| OsMPK17-2E1F | | GACTGAATTCGACATGGAGTTCTTTACGGAATACC |  |
| OsMPK17-2B1R | | GAGTGGATCCCTAGGAGAGCATCCTGGTCAC |  |
| OsMPK20-1E1F | | CAGAGAATTCATGCAGCAGGATCAGCGC |  |
| OsMPK20-1ClaR | | CAGCATCGATCTAATACATCCTCGACATGCCG |  |
| OsMPK20-2E1F | | CAGAGAATTCCGACGGTACTTGAGCAGCATGAGA |  |
| OsMPK20-2ClaR | | GTCAATCGATGCTGTTAGCCACTACTAATACATCCTTG |  |
| OsMPK20-3E1F | | CTCAGAATTCAAGATGCAGACCAGCAATTTTCG |  |
| OsMPK20-3BIR | | CTGTATCGATGGTCTAGGTGATTCTATACGTCCCT |  |
| OsMPK20-4ClaF | | GCATATCGATCGATGGCGATGCAGACGAT |  |
| OsMPK20-4B1R | | GACTGGATCCTAATACATCCTCGAGACCCCATAC |  |
| OsMPK20-5E1F | | CAGAGAATTCATGCCTGAGGCAAATGCGGGTG |  |
| OsMPK20-5BglR | | GTGCAGATCTAGTACATCCTTGAAACACCATATTGAAC |  |
| OsMPK21-1claF | | GACAATCGATACGAGGGCCGGTTTATGG |  |
| OsMPK21-1sacR | | GTGAGAGCTCGGGAATCATCAGGTTTTCAGTTG |  |
| OsMPK21-2E1F | | GACAGAATTCATGGATGCTAAGAAGGGCTC |  |
| OsMPK21-2BIR | | GTCGGGATCCGGATTGATCACCTGTACAGTAAGAGATAC |  |
| **Pull-down** | | |  |
| \| MKKK62-GSTF \| \| --- \| | \| TGTACAGGATCCTCCGAGGATGGCTGGAT \| \| --- \| | | |
| \| MKKK62-GSTR \| \| --- \| | \| CGGCGGGAATTCTTAAAAATTGCTCATTCTAGTCAAAACAGTG \| \| --- \| | | |
| \| MKK3-GSTF \| \| --- \| | GATCTGGTTCCGCGTGGATCCATGGCGGGGCTCGAGGAG | | |
| \| MKK3-GSTR \| \| --- \| | GTCACGATGCGGCCGCTCGAGTCAGGCTTGGATGATGTATAG | | |
| \| MKK3-HisF \| \| --- \| | CAGCAAATGGGTCGCGGATCCATGGCGGGGCTCGAGGAG | | |
| \| MKK3-HisR \| \| --- \| | GTGGTGGTGGTGGTGCTCGAGGGCTTGGATGATGTATAGATC | | |
| \| MKK10-2-HisF \| \| --- \| | CAGCAAATGGGTCGCGGATCCATGGCTCTCGTCCGCCAG | | |
| \| MKK10-2-HisR \| \| --- \| | GTGGTGGTGGTGGTGCTCGAGTAGATCGCCGCTCTGCTC | | |
| \| MAPK7-HisF \| \| --- \| | CAGCAAATGGGTCGCGGATCCATGGCGATGATGGTGGAC | | |
| \| MAPK7-HisR \| \| --- \| | GTGGTGGTGGTGGTGCTCGAGCATATTCACTCCTGCAACAAC | | |
| \| MAPK14-HisF \| \| --- \| | CAGCAAATGGGTCGCGGATCCATGGCGATCATGGTGGATC | | |
| \| MAPK14-HisR \| \| --- \| | GTGGTGGTGGTGGTGCTCGAGTCGGGCACTCATTGCTGCAAC | | |
| **Expressionanalysis** | | |  |
| MKKK62F | | CGATCATCGAGATGGCCACA |  |
| MKKK62R | | CGAGAAATGGGTGCTCCAGT |  |
| OsMFTF | | CACCCTGATCATGACGGACC |  |
| OsMFTR | | TACCTGTGTATCCCCACCGT |  |
| EF1α-F | | \| TTTCACTCTTGGTGTGAAGCAGAT \| \| --- \| |  |
| EF1α-R | | GACTTCCTTCACGATTTCATCGTAA |  |
