# Supplementary material for: The MKKK62-MKK3-MAPK7/14 module negatively regulates seed dormancy in rice
Source: Rice (N Y). 2019 Jan 22;12:2. doi: 10.1186/s12284-018-0260-z (PMC6342742; doi:10.1186/s12284-018-0260-z)
Supplement: Supplementary file 9 — Table S3. Characteristic fragment ions of hormone standards and optimized MS/MS conditions. (DOCX 15 kb) [file 12284_2018_260_MOESM9_ESM.docx]

Table S3. Characteristic fragment ions of hormone standards and optimized MS/MS conditions.

|  |  | Transition1 |  |  | Transition2 |  |  |
| --- | --- | --- | --- | --- | --- | --- | --- |
| Analyte | ESI mode | Quantitative  ion | Collision  energy/ev | Declustering potential/ev | Quantitative  ion | Collision  energy/ev | Declustering potential/ev |
| ABA | ESI- | 262.9>152.8 | -15 | -60 | 262.9>203.9 | -25 | -60 |
| GA3 | ESI- | 345>142.8 | -40 | -75 | 345>220.9 | -33 | -75 |
| GA4 | ESI- | 331>257.1 | -32 | -96 | 331>212.9 | -42 | -96 |
