# Supplementary material for: The MKKK62-MKK3-MAPK7/14 module negatively regulates seed dormancy in rice
Source: Rice (N Y). 2019 Jan 22;12:2. doi: 10.1186/s12284-018-0260-z (PMC6342742; doi:10.1186/s12284-018-0260-z)
Supplement: Supplementary file 5 [file 12284_2018_260_MOESM1_ESM.docx]

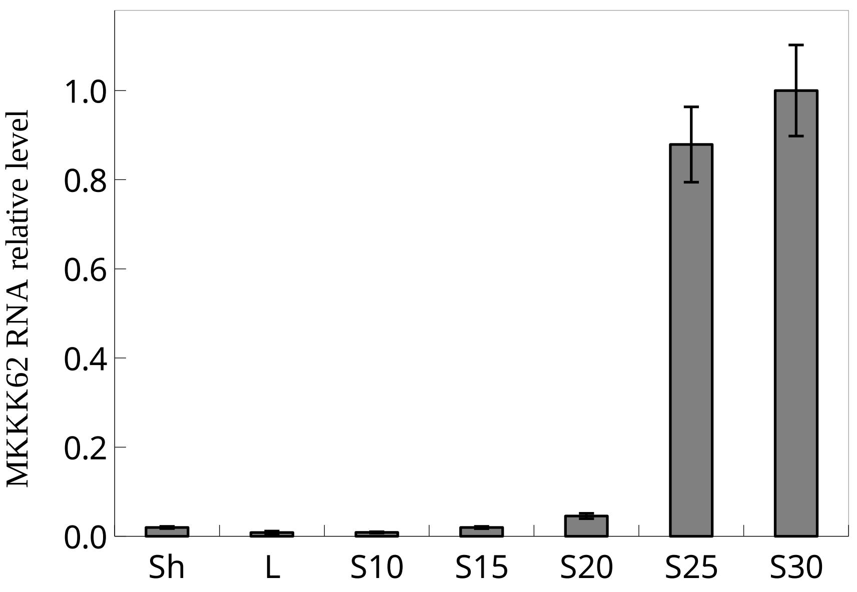

MKKK62 RNA relative level
1.0
0.8
0.6
0.4
0.2
0.0
Sh
L
S10
S15
S20
S25
S30
