# Supplementary material for: The MKKK62-MKK3-MAPK7/14 module negatively regulates seed dormancy in rice
Source: Rice (N Y). 2019 Jan 22;12:2. doi: 10.1186/s12284-018-0260-z (PMC6342742; doi:10.1186/s12284-018-0260-z)
Supplement: Supplementary file 6 [file 12284_2018_260_MOESM6_ESM.docx]

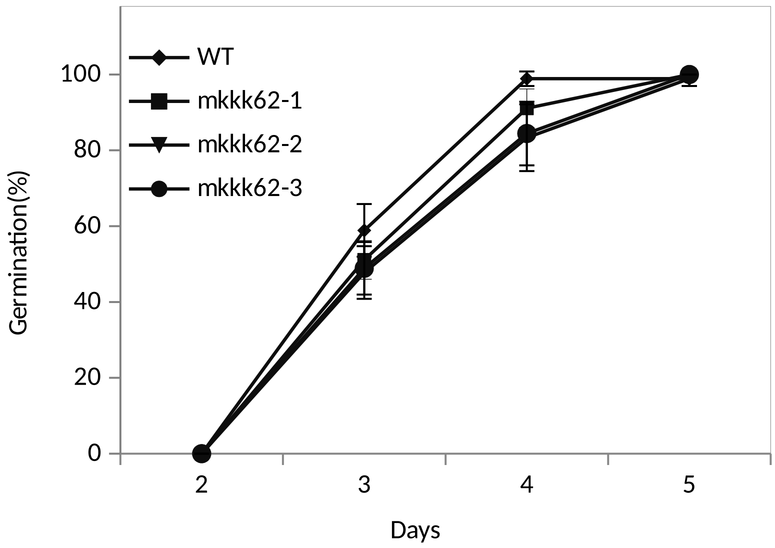
WT
mkkk62-1
mkkk62-2
mkkk62-3
Germination(%)
0
20
40
60
80
100
2
3
4
5
Days
